# HP1 cooperates with CAF-1 to compact heterochromatic transgene repeats in mammalian cells

**DOI:** 10.1038/s41598-018-32381-7

**Published:** 2018-09-20

**Authors:** Haiyan Yan, Xingfeng Xiang, Qinfu Chen, Xuan Pan, Hankun Cheng, Fangwei Wang

**Affiliations:** Life Sciences Institute and Innovation Center for Cell Signaling Network, Zhejiang University, Hangzhou, Zhejiang 310058 China

## Abstract

The nuclear organization of tightly condensed heterochromatin plays important roles in regulating gene transcription and genome integrity. Heterochromatic domains are usually present at chromosomal regions containing a large array of repeated DNA sequences. We previously showed that integration of a 1,000-copy tandem array of an inducible reporter gene into the genome of mammalian cells induces the formation of a highly compact heterochromatic domain enriched in heterochromatin protein 1 (HP1). It remains to be determined how these DNA repeats are packaged into a heterochromatic form and are silenced. Here, we show that HP1-mediated transgene condensation and silencing require the interaction with PxVxL motif-containing proteins. The chromatin assembly factor 1 (CAF-1) complex concentrates at the transgenic locus through the interaction of its PxVxL motif-containing p150 subunit with HP1. Knockdown of p150 relieves HP1-mediated transgene compaction and repression. When targeted to the transgenic locus, p150 mutants defective in binding HP1 cause transgene decondensation and activation. Taken together, these results suggest that HP1 cooperates with CAF-1 to compact transgene repeats. This study provides important insight into how heterochromatin is maintained at chromosomal regions with abundant DNA repeats.

## Introduction

The organization and regulated expression of the large eukaryotic genome requires sophisticated packaging of DNA into the tiny space of nucleus^1^. The genomic DNA in a single human cell, stretching to nearly 2.0 meters in length if attached end to end, wraps with histones to form nucleosome, the basic unit of chromatin. Nucleosomes are further packaged into higher-order chromatin structures to form distinctive domains of euchromatin and heterochromatin. Heterochromatin, a tightly packed form of DNA, is usually found in chromosomal regions containing a high density of repetitive DNA sequences such as transposons and satellite DNA^2^, and plays essential roles in maintaining epigenetic gene silencing and genome stability.

Heterochromatin also assembles at transgene repeats, generally resulting in transcriptional transgene silencing. Studies in a variety of organisms suggest a universal phenomenon that repetitive transgene can be sufficient for inducing heterochromatin formation^3,4^. The formation of repressive heterochromatin at transgene repeats may reflect a cellular defense mechanism against the invasion of these threatening sequence elements. However, the mechanism for heterochromatinization at transgene repeats remains elusive.

**Figure 1:**
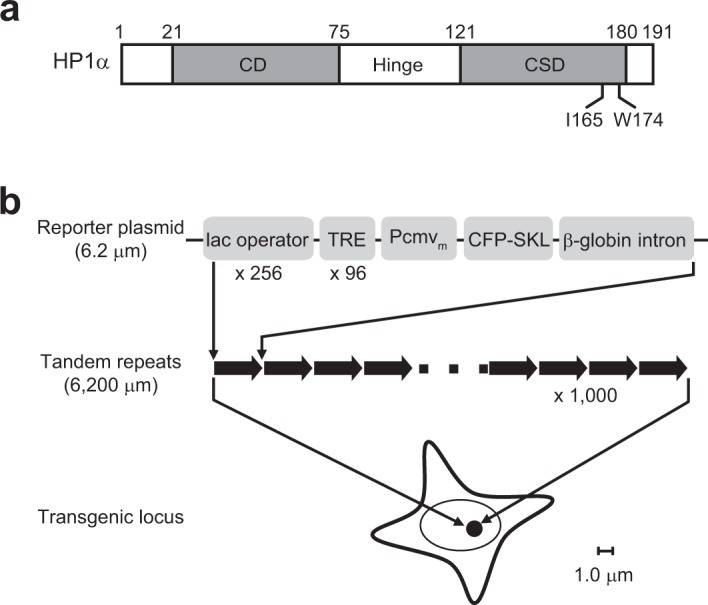
Schematics of human HP1α and the transgene array in clone 2 of BHK cells. (**a**) Human HP1α consists of an N-terminal CD and a C-terminal CSD linked by a flexible hinge region. The I165E mutation eliminates CSD self-dimerization and the binding to proteins that require a dimerized CSD, whereas the W174A mutation retains the dimerization but eliminates binding to PxVxL-containing proteins. (**b**) Clone 2 cells with a 1,000-copy inducible reporter plasmid tandemly integrated into a single site in the genome. The reporter gene was constructed in the pBluescriptIIKS(−) plasmid. It is composed of 256 copies of the lac operator sequence followed by 96 copies of TRE controlling a CMVm promoter which regulates the expression of CFP-SKL targeted to peroxisomes. Note that the rest of pBluescriptIIKS(−) is not shown.

As a hallmark of heterochromatin, heterochromatin protein 1 (HP1) plays an critical role in heterochromatin formation and gene silencing^5^. HP1 consists of an N-terminal chromodomain (CD) and a C-terminal chromo-shadow domain (CSD) linked by a flexible hinge region containing a nuclear localization signal (NLS) (Fig. 1a). The CD binds to di- or tri-methylated lysine 9 of histone H3 (H3K9me2/3) created by histone methyltransferase (HMT)^6–9^, whereas the CSD functions as a dimerization module^10,11^ and mediates interactions with a variety of nuclear proteins. HP1 is thought to act as a structural adaptor by bringing together different proteins to the targeted region to fulfill its various duties^12^. The HP1 CSD-interacting proteins typically contain a pentapeptide motif PxVxL (x represents any amino acid), such as the p150 subunit of chromatin assembly factor 1 (CAF-1)^13,14^. The three-subunit complex (p150, p60 and p48) of CAF-1 is a histone chaperone responsible for depositing newly synthesized histones H3 and H4 into nascent chromatin during DNA replication^15,16^. CAF-1/p150-HP1 interaction is required for pericentromeric heterochromatin replication in S-phase and also plays a role in DNA damage responses^17–19^.

Tsukamoto *et al*. previously developed a system for visualizing gene activity in living cells^20^. This system consists of a stable cell line (“clone 2”) in which a 1,000-copy inducible reporter plasmid was tandemly integrated into a single site in the genome of baby hamster kidney (BHK) cells (Fig. 1b). The 18.52-kilobase-long reporter plasmid is composed of 256 copies of the lac operator repeats, followed by 96 copies of a tetracycline-responsive element (TRE) controlling a minimal CMV promoter (CMVm) that regulates the expression of a cyan fluorescent protein (CFP) with a peroxisome targeting signal SKL (serine-lysine-leucine tripeptide). Upon transcriptional induction by doxycycline (Dox), Tet-On-VP6, a fusion protein of reverse-tet repressor (rTetR) and the VP16 acidic activation domain (AAD), is recruited to the TRE repeats and subsequently induces transgene activation. Reporter gene expression is monitored by the appearance of peroxisome-targeted CFP signals, accompanied by decondensation of the transgene visualized by binding of a fluorescently tagged Lac repressor (lacR) to the lac operator repeats. The un-induced transgene remains highly condensed throughout the cell cycle and is rich in H3K9me3 and HP1^21^. The 1,000-copy transgene array extends over a chromosome region of nearly 20 Mb in length, around 6,000 µm if stretched out. How such a long repetitive transgene array is compacted into a highly condensed chromatin domain of close to 1.0 µm in diameter remains unknown.

This cell system, in which an inducible transgene is embedded in repetitive sequences, is unique in the way that it allows precisely controlled transcriptional induction, direct visualization, targeted manipulation and simultaneous analysis of chromatin structure and gene expression. It thus provides a powerful system to investigate how DNA repeats are packaged into a heterochromatic domain and are silenced. Utilizing this system, we demonstrate that HP1 cooperates with CAF-1 to compact transgene repeats in a heterochromatic domain in mammalian cells.

## Results

### HP1 mutants incapable of binding PxVxL motif-containing proteins are defective in inducing transgene silencing and condensation

**Figure 2:**
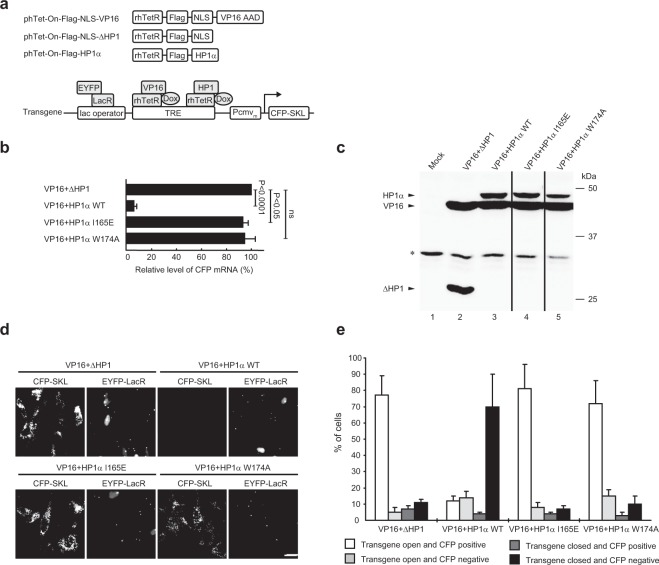
HP1 mutants incapable of binding PxVxL motif-containing proteins are defective in inducing transgene silencing and condensation. Cells were cotransfected with plasmids as indicated, the expression and morphology of the transgene were analyzed. (**a**) Schematics show the expression plasmids, and simultaneous targeting of VP16 and HP1α to the TRE sequence of the transgene in the presence of Dox. (**b**) Real-time RT-PCR analysis of CFP mRNA levels. The relative CFP mRNA level was determined by normalizing the ratio of CFP mRNA to β-actin mRNA upon cotransfection with VP16 and HP1α against that in cells expressing VP16 and ΔHP1. VP16 represents phTet-On-Flag-NLS-VP16; ΔHP1 represents phTet-On-Flag-NLS-ΔHP1; HP1α represents phTet-On-Flag-HP1α. Means and standard deviations (SDs) are shown (n = 3; un-paired *t*-test). (**c**) Immunoblotting of transfected proteins using the anti-Flag antibody. Irrelevant lanes were removed between lanes 3 and 4, 4 and 5. Asterisk points to non-specific bands. The original full-length blots are shown in Supplementary Fig. S1 in the Supplemental Information. (**d**) Fluorescence microscopy of transgene expression, which is monitored by the peroxisomal CFP-SKL signals in the cytoplasm. The transgenic locus is visualized by binding of EYFP-LacR to the lac operator repeats. Scale bar, 20 µm. (**e**) Classification of transgene morphology and CFP signals as indicated. The heterogeneous and extended arrays are considered “open”, whereas the homogeneous and compact arrays are considered “closed”. Means and ranges from two independent experiments are shown (n = 200).

Tethering HP1 to the transgenic locus in clone 2 cells simultaneously suppressed VP16-induced transgene activation and decondensation^21^, which prompted us to examine the underlying mechanism. As shown in Fig. 2a, HP1α cDNAs with mutations in the CSD were inserted into the phTet-On-Flag vector encoding a Flag-tagged humanized rTetR (hrTetR), resulting in phTet-On-Flag-HP1α. Clone 2 cells were cotransfected with phTet-On-Flag-HP1α and phTet-On-Flag-NLS-VP16, which encodes a Flag-tagged fusion protein consisting of hrTetR, NLS and VP16 AAD. A plasmid, phTet-On-Flag-NLS (ΔHP1 in short), encoding a Flag-tagged hrTetR with NLS was used as a control. pEYFP-LacR encoding an enhanced yellow fluorescence protein (EYFP)-fused LacR was also cotransfected to visualize the transgenic locus. After incubation for 18 hours in the presence of Dox, the effects of targeted HP1 on VP16-induced transgene activation and decondensation were examined as we previously described^21^.

RT-PCR analysis showed that targeting of wild-type (WT) HP1α significantly suppressed VP16-induced transgene activation, as evidenced by a 16.7-fold reduction in the CFP mRNA levels compared to control cells (Fig. 2b). In contrast, targeting of HP1αI165E which is incapable of self-dimerization and interaction with proteins that require dimerization^22^, failed to prevent the transgene activation. Similar results were obtained with the HP1αW174A mutant, which retains the dimerization activity but is defective in interacting with proteins which typically contain a PxVxL motif^22^. Immunoblotting confirmed that VP16 was expressed at similar levels among the samples and that HP1α mutants were expressed at comparable levels to WT HP1α (Fig. 2c, Fig. S1). Consistently, fluorescence microscopy showed that the peroxisomal CFP signals were much lower in WT HP1α-targeted cells than in cells targeted by the mutant of I165E or W174A (Fig. 2d), confirming that these two mutants are defective in suppressing transgene expression.

Moreover, in control cells, 77% of cells (n = 200) showed a decondensed transgenic locus with peroxisomal CFP signals. In contrast, upon targeting of HP1α, only 12% of cells behaved so, while a large proportion of cells (70%) exhibited a closed locus without peroxisomal CFP signals (Fig. 2e). This indicates that targeting of HP1α simultaneously repressed transgene expression and decondensation, as we previously showed^21^. Similar to control cells, targeting of HP1αI165E and HP1αW174A led to 81% and 72% of cells showing a decondensed transgene array with peroxisomal CFP signals, respectively. This indicates that HP1αI165E and HP1αW174A are defective in antagonizing VP16-induced transgene transcription and decondensation. Taken together, these results suggest that CSD-dependent interaction with proteins containing the PxVxL motif is necessary for HP1-mediated transgene silencing and compaction.

### CAF-1 concentrates at the transgenic locus through the interaction of p150 with HP1

To understand how HP1 promotes heterochromatinization of the transgene repeats, we focused on p150, a PxVxL motif-containing subunit of CAF-1 which associates with endogenous heterochromatin through binding the CSD of HP1^13,18,23^. We first examined whether CAF-1 is associated with the condensed transgenic locus. Enhanced CFP-fused individual subunits of CAF-1 (ECFP-p150, ECFP-p60 and ECFP-p48) were transiently transfected with pEYFP-LacR into cells and their nuclear distribution pattern were examined by fluorescence microscopy.

**Figure 3:**
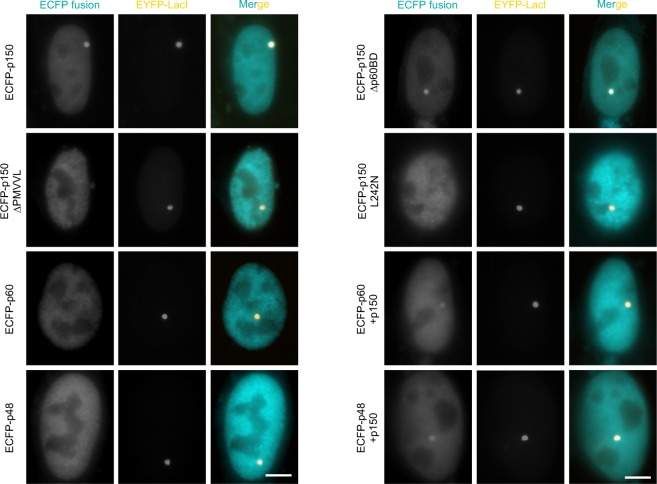
CAF-1 concentrates at the transgenic locus through the interaction between p150 and HP1. Cells were cotransfected with pEYFP-lacR and plasmids encoding ECFP-fused CAF-1 subunits as indicated. The nuclear localization of ECFP-fusion proteins relative to the transgenic locus marked by EYFP-LacR was examined by fluorescence microscopy. Note that co-expression of exogenous un-tagged p150 facilitates concentration of ECFP-p60 and ECFP-p48 at the transgenic locus. Scale bar, 5 µm.

As shown in Fig. 3, concentrated localization of ECFP-p150 was detected at the transgenic locus marked by EYFP-LacR. Moreover, ECFP-p150Δp60BD, which lacks the p60 binding domain (p60BD encompassing residues 642–677)^16^, retained the ability to localize to the transgene array. This indicates that interaction with p60 is not necessary for the association of p150 with the transgene array. In contrast, ECFP-p150ΔPMVVL lacking the consensus PxVxL motif (residues 238–242) and ECFP-p150L242N with a point mutation L242N in the motif were defective in localizing to the transgene array in around 82% of interphase cells (n = 100). Since p150ΔPMVVL and p150L242N are defective in interacting with HP1^13^, these results indicate that p150 is enriched at the transgene array through binding HP1. ECFP-p60 and ECFP-p48 only weakly associated with the transgenic locus. However, co-expression of exogenous p150 encoded by pSV2-p150 strongly increased the localization of ECFP-p60 and ECFP-p48 at the transgenic locus, suggesting that the association of p60 and p48 with the transgene array is p150-dependent. Taken together, CAF-1 complex concentrates at the transgene array through the interaction between p150 and HP1.

### Knockdown of p150 diminishes HP1-mediated transgene silencing and compaction

**Figure 4:**
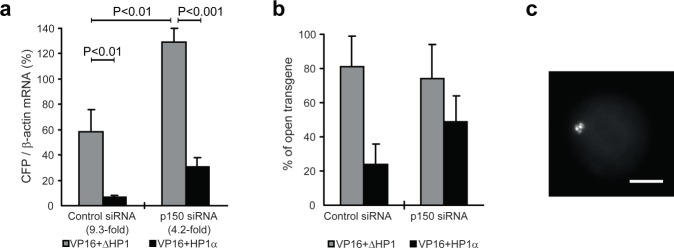
Knockdown of p150 diminishes HP1-mediated transgene silencing and compaction. Cells were depleted of p150 by RNAi followed by plasmid transfection as described in Fig. 2a, and the expression and morphology of the transgene were analyzed. (**a**) RT-PCR analysis of the relative CFP mRNA level defined as the ratio to β-actin mRNA. The repression fold shown in the parentheses was determined by normalizing the ratio of CFP mRNA/β-actin in cells expressing VP16 and ΔHP1 to that in cells expressing VP16 and HP1α. Means and SDs are shown (n = 3; un-paired *t*-test). (**b**) Percentage of cells with decondensed transgene array based on fluorescence microscopy. Means and ranges from two independent experiments are shown (n = 200). VP16 represents phTet-On-Flag-NLS-VP16; ΔHP1 represents phTet-On-Flag-NLS-ΔHP1; HP1α represents phTet-On-Flag-HP1α. (**c**) Example image of a cell with an open transgene locus.

We next determined whether p150 contributes to HP1-mediated transgene silencing and condensation in the assay described in Fig. 2a. Following depletion of p150 by RNA inteference (RNAi), phTet-On-Flag-HP1α (or phTet-On-Flag-NLS as a control) was cotransfected into cells with phTet-On-Flag-NLS-VP16 and pEYFP-LacR, and the transgene expression was analyzed as described above. RT-PCR analysis revealed that p150 was depleted by 68%-75% at the mRNA level. The effect of p150 depletion on transgene activation was determined by the relative mRNA levels of CFP to β-actin. As shown in Fig. 4a, targeting of HP1α caused a 9.3-fold reduction in the CFP mRNA level in control siRNA-transfected cells. In contrast, when p150 was depleted, HP1a targeting achieved only a 4.2-fold reduction, indicating that HP1-mediated transgene silencing was diminished by p150 RNAi.

Fluorescence microscopy showed that, in control RNAi cells, targeting of HP1α resulted in a decrease in the percentage of cells with a VP16-induced decondensed transgene array from 81% to 23% (Fig. 4b,c). Interestingly, p150 depletion clearly impaired this HP1α-mediated transgene condensation, as evidenced by 57% of cells showing a decondensed transgene array. Thus, p150 contributes to HP1-mediated transgene silencing and compaction.

### Knockdown of p150 promotes transgene activation

The requirement of p150 for HP1-mediated transgene heterochromatinization prompted us to examine the effect of p150 depletion on condensation of the transgene array. Cells were subjected to three consecutive siRNA transfections followed by transfection with pEYFP-LacR to visualize the transgenic locus. Fluorescence microcopy showed that, in almost all cells transfected with control siRNA, the transgenic locus was compact and homogenous in morphology. In contrast, 26% of p150-depleted cells showed a still compact yet heterogeneous transgenic locus (n = 100), suggesting that p150 depletion might relieve compact state of the transgene array in a fraction of cells.

Chromatin unfolding generally increases the accessibility of the embedded gene to transcription activators and promotes gene expression. We therefore examined whether p150 depletion induces transgene expression without transfection of a transcription activator. RT-PCR analysis did not detect CFP mRNA in p150-depleted cells as in control RNAi cells, suggesting that p150 knockdown alone does not directly induce transgene expression in the absence of an exogenous activator.

**Figure 5:**
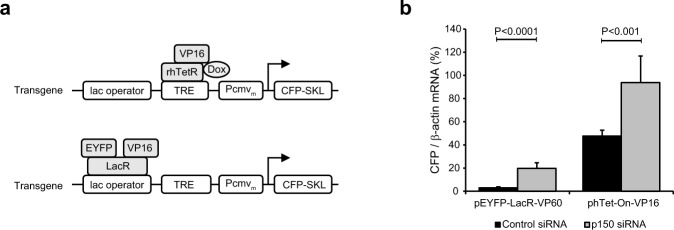
Knockdown of p150 promotes the transgene activation. Cells were depleted of p150 by RNAi followed by transfection with plasmids as indicated. (**a**) Induction of the transgene expression by binding of the transcriptional activator encoded by phTet-On-VP16 or pEYFP-LacR-VP16. (**b**) Transgene activation was determined by RT-PCR analysis of the ratio of CFP mRNA to β-actin mRNA. The up-regulation fold shown in the parentheses was determined by normalizing the ratio of CFP mRNA/β-actin in p150-depleted cells to that in corresponding control RNAi cells. Means and SDs are shown (n = 3; un-paired *t*-test).

We next examined the effect of p150 RNAi on transgene activation induced by transiently transfected activators. For this purpose, following p150 RNAi, cells were transfected with plasmids expressing transcription activator targeted to different sequences of the transgene array (Fig. 5a). RT-PCR analysis showed that the CFP mRNA level in p150-depleted cells was 2.0-fold higher than that in control RNAi cells when the transgene was activated by hTet-On-VP16 (Fig. 5b, see also gray bars in Fig. 4a). Thus, p150 RNAi up-regulates the transgene activation induced by VP16 targeted to the TRE repeats.

The lac operator repeats represent a larger proportion of the transgene array than the TRE repeats. We therefore examined how p150 knockdown affects transgene expression by VP16 targeted to the lac operator repeats. In control RNAi cells, the transgene was 24.6-fold less activated by EYFP-LacR-VP16 than by hTet-On-VP16, probably due to the distance between LacR-VP16 and CMV promoter. No obvious peroxisomal CFP signals were detected in cells transfected with EYFP-LacR-VP16, though mild transgene unfolding was observed. Thus, VP16 is less potent in inducing the transgene expression when targeted to the lac operator repeats. Importantly, upon induction by EYFP-LacR-VP16, the CFP mRNA level in p150-depleted cells was 6.8-fold higher than that in control RNAi cells (Fig. 5b). Taken together, p150 knockdown causes up-regulation of transgene expression regardless of the sequence to which the activator is targeted.

### Targeting of p150 mutants defective in HP1 interaction causes large-scale decondensation of the transgene array

Up-regulation of transgene activation upon p150 knockdown suggests a contribution of p150 to transgene condensation. To further corroborate this speculation, we sought an alternative approach by targeting p150 to the transgene array. For this purpose, different forms of p150 as fusion proteins with EYFP-LacR were targeted to the lac operator repeats of the transgene, and the morphology of the transgenic locus was examined by fluorescence microscopy.

**Figure 6:**
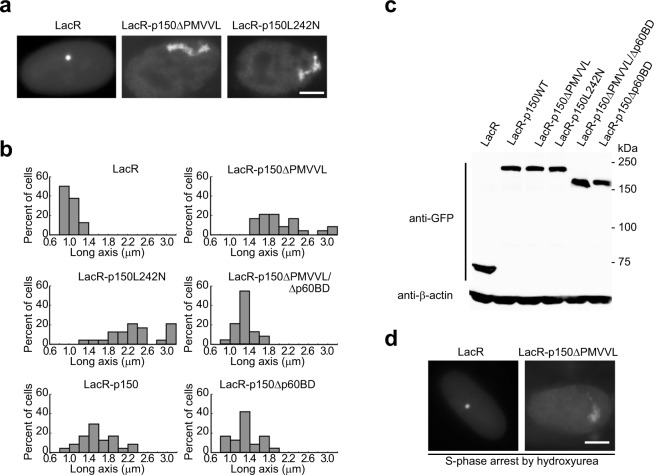
Targeting of p150 mutants defective in HP1 interaction leads to large-scale decondensation of the transgene array. (**a**) Cells were transfected with plasmids as indicated, and the transgene morphology was analyzed by fluorescence microscopy. Example images of cells transfected with plasmids encoding EYFP-LacR, EYFP-LacR-p150ΔPMVVL or EYFP-LacR-p150L242N. (**b**) Quantitative measurements of the long axis of transgene arrays marked by EYFP-LacR fusion proteins (n = 24). The bars corresponding to over 3 µm in long axis represent the percentage of transgene arrays larger than 3 µm. (**c**) Immunoblotting of EYFP-LacR fusion proteins using the anti-GFP antibody, with endogenous β-actin as a loading control. (**d**) Cells were transfected with the plasmid encoding EYFP-LacR or EYFP-LacR-p150ΔPMVVL. At 4 hours after transfection, cells were treated with hydroxyurea (2 mM) for 24 hours, then the transgene morphology was analyzed by fluorescence microscopy. Scale bar, 5 µm.

Representative images of the cells transfected with pEYFP-LacR, pEYFP-LacR-p150ΔPMVVL or pEYFP-LacR-p150L242N are shown in Fig. 6a. To quantify the size of the transgene array upon targeting of the various proteins, the long axis of randomly selected EYFP-LacR-labeled transgene arrays were measured (n = 24). As shown in Fig. 6b, control cells expressing EYFP-LacR showed a highly condensed transgenic locus with a mean long axis of 1.01 ± 0.15 µm. The transgene in cells expressing EYFP-LacR-p150 (1.61 ± 0.41 μm) was larger than that in control cells, but remained condensed in cells with low to moderate levels of EYFP-LacR-p150. Moderately unfolded transgene was found only in cells with high levels of EYFP-LacR-p150. Interestingly, the transgene size in cells expressing EYFP-LacR-p150ΔPMVVL (2.03 ± 0.51 µm) or EYFP-LacR-p150L242N (2.43 ± 0.69 µm) was significantly larger than that in control cells regardless of the expression level. Thus, targeting of p150ΔPMVVL or p150L242N defective in HP1 interaction causes strong decondensation of the transgene array. No significant transgene decondensation was observed in cells expressing EYFP-LacR-p150ΔPMVVL/Δp60BD (1.29 ± 0.31 µm). This double mutant lacks interaction with both HP1 and p60, suggesting that binding to p60 is necessary for the p150ΔPMVVL-induced transgene decondensation. The transgene in cells expressing EYFP-LacR-p150Δp60BD (1.30 ± 0.19 µm) lacking interaction with p60 was slightly smaller than that in cells expressing EYFP-LacR-p150. Moreover, 20.8% and 54.2% of cells expressing EYFP-LacR-p150ΔPMVVL and EYFP-LacR-p150L242N exhibited a highly decondensed transgene array (>2.4 μm in long axis), respectively, whereas no transgene over 2.4 μm in long axis was observed in cells expressing WT p150 or other p150 mutants. Immunoblotting using anti-GFP antibodies confirmed the similar expression levels of these EYFP-LacR-fused proteins (Fig. 6c).

Given the role of CAF-1/p150 in chromatin replication in S-phase, we next examined whether the transgene decondensation induced by p150ΔPMVVL targeting requires passage through S-phase. To this purpose, we treated EYFP-LacR-p150ΔPMVVL-expressing cells with hydroxyurea, which blocks cells in S-phase by inhibiting the production of dNTPs. Fluorescence microscopy showed that expression of EYFP-LacR-p150ΔPMVVL, but not EYFP-LacR, caused strong decondensation of the transgene array in S-phase cells as in asynchronous cell (Fig. 6d).

Taken together, these results show that targeting of a mutant CAF-1 lacking binding to HP1 induces large-scale decondensation of the transgene array.

### Delocalization of HP1β and H3K9me3 from the decondensed transgene array bound by the HP1-binding-deficient mutant of p150

**Figure 7:**
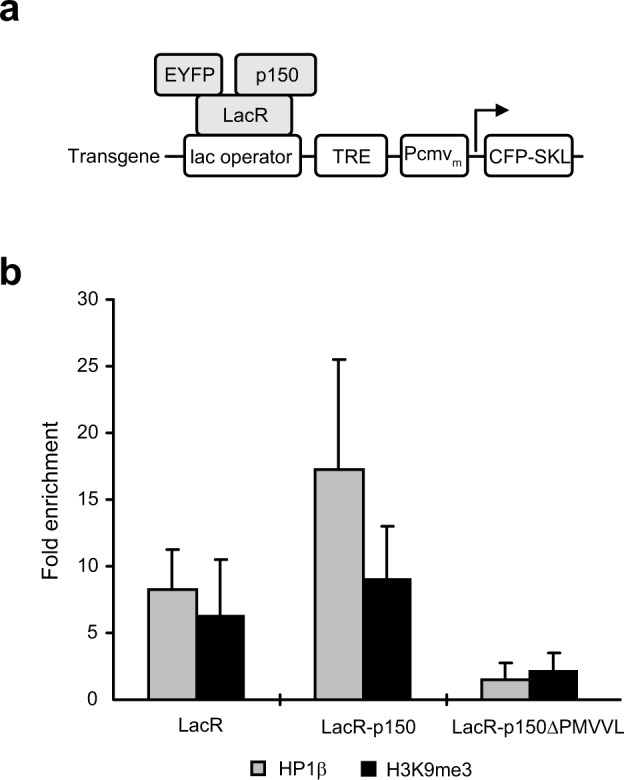
Delocalization of HP1β and H3K9me3 from the decondensed transgene array bound by the HP1-binding-deficient mutant of p150. Cells were transfected with plasmids encoding EYFP-LacR fusion proteins as shown in (**a**) followed by ChIP assay using antibodies specific for HP1β or H3K9me3 (**b**). The fold enrichment was determined by normalizing the immunoprecipitation percentage of transgene sequences (CMVm promoter), defined as the ratio of immunoprecipitated DNA to input DNA, against that of the active ß-actin promoter region. Means and ranges from two independent experiments are shown.

Decondensation of the transgenic locus induced by targeted p150ΔPMVVL and p150L242N suggests the importance of HP1-p150 interaction for transgene compaction. We therefore examined the association of HP1 with the decondensed transgene array by chromatin immunoprecipitation (ChIP) assay. Following transfection with pEYFP-LacR, pEYFP-LacR-p150 or pEYFP-LacR-p150ΔPMVVL (Fig. 7a), cells were subjected to ChIP assay using HP1ß specific antibody as we previously reported^21^. As expected, cells expressing EYFP-LacR showed an 8.2-fold enrichment of HP1ß at the CMVm promoter region of the transgene relative to the active ß-actin promoter region (Fig. 7b). Upon expression of EYFP-LacR-p150, the enrichment of HP1ß was increased to 17.3-fold, indicating the recruitment of endogenous HP1ß to the transgene array. Moreover, a moderate increase in H3K9me3 from 6.3-fold in cells expressing EYFP-LacR to 9.0-fold was detected in cells expressing EYFP-LacR-p150, suggestive of recruitment of an H3K9 HMT. Importantly, in cells expressing EYFP-LacR-p150ΔPMVVL, the enrichment of HP1ß and H3K9me3 at the transgene array were reduced to 1.5- and 2.1-fold, respectively. Taken together, these results show that decondensation of the transgene array caused by targeted p150ΔPMVVL is accompanied by loss of HP1 and H3K9me3.

### Targeting of p150 mutants defective in HP1 interaction promotes the transgene activation

Chromatin decondensation is often accompanied by increased gene expression. We therefore tested whether targeting of p150 affects transgene expression. We first determined whether targeting of EYFP-LacR-p150 to the transgenic locus induces transgene expression. Similar to that in mock-transfected cells, RT-PCR analysis did not show detectable CFP mRNA in cells expressing EYFP-LacR, EYFP-LacR-p150 or EYFP-LacR-p150ΔPMVVL. This indicates that, when targeted to the lac operator sequences, the WT p150 does not directly induce transgene expression and that massive transgene decondensation caused by EYFP-LacR-p150ΔPMVVL is not accompanied with transgene activation in the absence of a transfected activator.

**Figure 8:**
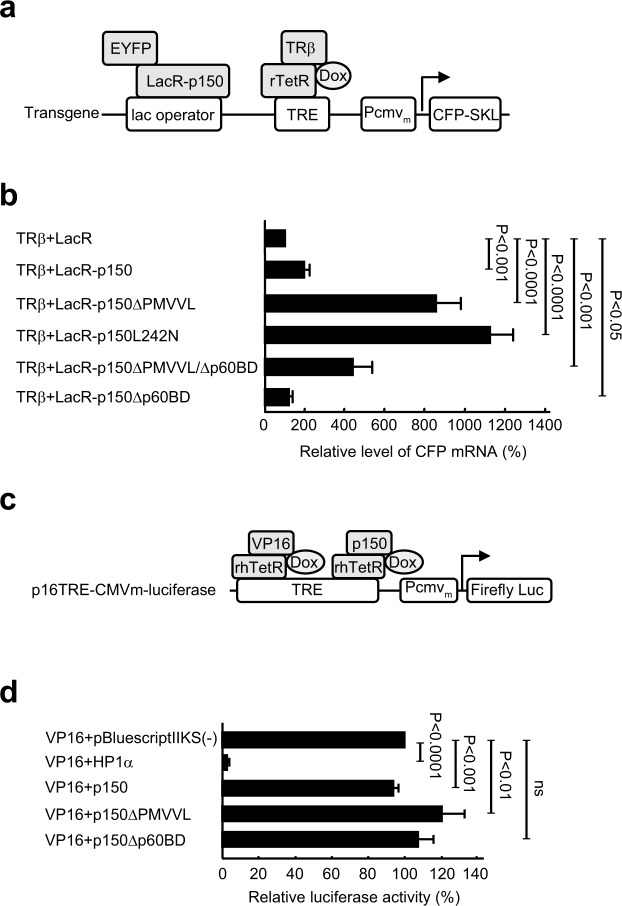
Targeting of p150 mutants defective in HP1 interaction up-regulates the transgene activation. (**a**) Clone 2 cells were cotransfected with pTet-On-TR and pEYFP-LacR fusion constructs as indicated. Upon induction by Dox and T3, TRβ is targeted to the TRE repeats and activates transgene activation. (**b**) The effect of targeted p150 on TRβ-induced transgene expression was determined by RT-PCR analysis of the CFP mRNA levels. The relative CFP mRNA level was determined by normalizing the CFP mRNA/β-actin mRNA ratio to that in induced control cells cotransfected with pTet-On-TR and pEYFP-LacR. Means and SDs are shown (n = 3; un-paired *t*-test). (**c**) Parental BHK cells were cotransfected with p16TRE-CMVm-luciferase, phRL-TK, phTet-On-Flag-NLS-VP16 and phTet-On-Flag-p150 as indicated. The reporter firefly luciferase gene was activated by the targeted VP16. (**d**) Dual luciferase analysis of the relative firefly luciferase activity, determined by normalizing the ratio of firefly luciferase activity to *Renilla* luciferase activity against that in cells cotransfected with phTet-On-Flag-NLS-VP16 and pBluescriptIIKS(−). Means and SDs are shown (n = 6; un-paired *t*-test).

We next examined whether targeting of p150 affects the transgene activation induced by an exogenously expressed activator. For this purpose, cells were cotransfected with pEYFP-LacR fusion constructs and pTet-On-TR, which encodes the rTetR-fused transcription activator human thyroid hormone receptor β (TRβ), followed by incubation in the presence of Dox and triiodothyronine (T3) (Fig. 8a). TRβ was used as the activator here because it is a much weaker transcriptional activator than VP16, and targeting of TRβ itself does not cause detectable transgene decondensation. Therefore, the difference in TRβ-induced transgene expression would be correlated well with the extension of transgene decondensation.

RT-PCR analysis showed that, compared to the control cells targeted by EYFP-LacR, tethering EYFP-LacR-p150 only slightly increased the CFP mRNA level by 1.8-fold (Fig. 8b). This probably reflects the mild transgene unfolding in a fraction of cells highly expressing EYFP-LacR-p150. In contrast, targeting of p150ΔPMVVL and p150L242N significantly increased the CFP mRNA levels by 8.3- and 11.1-fold, respectively, consistent with the massive transgene decondensation induced by these two mutants. As expected from the defect of p150Δp60BD in unfolding the transgenic locus, targeting of p150Δp60BD had little effect on transgene activation. Taken together, transgene activation is significantly up-regulated when large-scale decondensation is induced by p150 mutants defective in HP1 interaction.

### Targeting of p150 does not repress the expression of a transient reporter gene

We next examined whether p150 (WT or mutants) can directly regulate transient gene expression. For this purpose, we used a reporter plasmid, p16TRE-CMVm-luciferase, which encodes a firefly luciferase gene regulated by the CMVm promoter downstream of 16-copies of the TRE repeat (Fig. 8c). This reporter plasmid and phTet-On-Flag-NLS-VP16 were cotransfected into the parental BHK cells with phTet-On-Flag fusion constructs, using the *renilla* luciferase expressing plasmid phRL-TK as an internal control. Both VP16 and p150 were simultaneously targeted to the TRE repeats in the presence of Dox, and the effect of p150 on VP16-induced reporter gene expression was determined by dual luciferase assay.

As expected, targeting of HP1α caused a 45.3-fold reduction in the firefly luciferase activity compared to control cells cotransfected with phTet-On-Flag-NLS-VP16 and pBluescriptIIKS(−) (Fig. 8d). In contrast, little effect on luciferase gene expression was observed upon targeting of WT p150 or p150Δp60BD. This indicates that p150 does not have intrinsic transcriptional repression activity toward a transient reporter gene. Moreover, only slight (1.2-fold) increase in the luciferase activity was achieved by targeting of p150ΔPMVVL. Taken together, these forms of p150 do not obviously affect the expression of a transient reporter gene in the absence of a genomic chromatin environment.

## Discussion

The application of the lac operator/repressor system for *in vivo* localization of DNA sequences and visualization of chromatin has tremendously facilitated the structural and functional analysis of higher-order chromatin organization and nuclear architecture^24,25^. By artificial targeting of a variety of proteins to the transgene array via lac operator/repressor or Tet operator/repressor binding, cells lines similar to the one used in this study have proven to be useful for revealing the physiological functions of many proteins *in vivo*^26,27^. Moreover, formation of condensed chromatin domain upon insertion of multicopy transgene was also reported in a number of different cell lines^28–31^. For example, when a 200-copy reporter plasmid integrates into a euchromatic region of U2OS cells, it forms a highly condensed heterochromatic domain in interphase cells^29^. These results suggest that heterochromatinization at transgene repeats is a universal phenomenon. In this study, we have shown that HP1 and CAF-1 work cooperatively to maintain compaction of a heterochromatic domain at a repetitive transgene array, providing a first link between HP1, CAF-1 and transgene heterochromatinization in mammals.

We first showed that, when forced targeted to the transgenic locus, HP1 is able to antagonize VP16-induced transgene activation and decondensation, suggesting the involvement of HP1 in maintaining the transgene compaction. The defect of HP1W174A mutant (which can dimerize but not bind PxVxL proteins) in inducing heterochromatinization suggests that self-dimerization of HP1 is insufficient to physically compact chromatinized DNA repeats. This is further supported by the impaired HP1-induced transgene heterochromatinization upon knockdown of p150, which concentrates at the transgene via HP1-binding. It is noteworthy that the mutation of W174A designed to eliminate HP1 interaction with PxVxL-containing proteins leads to more significant defect of HP1 activity than that achieved by p150 RNAi. This suggests that either p150 depletion by RNAi is not functionally sufficient, and/or other PxVxL-containing proteins also contribute to HP1-induced heterochromatic transgene silencing.

The organization of higher-order chromatin structures is tightly related to gene expression. Generally, condensed chromatin limits the accessibility of an embedded gene to transcriptional activators whereas decondensation renders it more competent for activation. To evaluate the contribution of CAF-1 to condensation of the transgene array, we found that p150 RNAi promotes activation of the transgene. The change in morphology of the transgenic locus, from round and homogenous to irregular and heterogeneous, in a small fraction of p150-depleted cells, is suggestive of some local chromatin unfolding. However, p150 RNAi did not cause obvious decondensation of the transgenic locus in the majority of p150-depleted cells. An obvious explanation is due to incomplete knockdown of p150. Indeed, full knockdown of p150 might be expected to cause S-phase arrest and lead to cell death^32^, and therefore make it impracticable to analyze the behavior of the transgene array in cells completely depleted of p150. Since p150 does not show intrinsic transcription repression activity toward a transient reporter gene, up-regulation of transgene activation upon p150 RNAi could be due to relaxation of the compact nature of the transgene array, which consequently facilitates transgene activation.

Strong evidence in support of the role of the HP1-CAF-1 complex in maintaining transgene condensation comes from the observation that targeting of p150 mutants (ΔPMVVL and L242N) defective in HP1 binding causes massive transgene decondensation accompanied by depletion of H3K9me3 and HP1. Moreover, up-regulation of transgene induction by the same activator (i.e. TRβ) is more significant when targeting EYFP-LacR-p150L242N to the locus (11.1-fold) than when p150 is depleted (3.0-fold). This is consistent with the large-scale transgene decondensation observed upon targeting of p150 mutants (ΔPMVVL and L242N) but not in p150-depleted cells. Notably, targeting of p150ΔPMVVL alone does not induce transgene expression in the absence of an exogenous activator. It therefore appears that transgene decondensation correlates with the potential for gene activation, rather than with gene transcription itself. Furthermore, p150ΔPMVVL/Δp60BD lacking interaction with both HP1 and p60 does not cause obvious transgene decondensation, suggesting that interaction with p60 is required for p150ΔPMVVL-induced transgene decondensation. This is different from the p60-independent role of p150 in pericentric heterochromatin replication reported in mouse cells^17^, and is consistent with our observation that DNA replication inhibitors do not prevent transgene decondensation induced by EYFP-LacR-p150ΔPMVVL. We therefore reason that the p150 mutants of ΔPMVVL and L242N could titrate the p60 and p48 subunits into a non-functional HP1-binding deficient CAF-1 complex, which competes with the endogenous counterpart, resulting in compromised HP1 recruitment. Because H3K9 HMTs can bind HP1, this might also explain the observed decline in H3K9me3 at the locus. Together, these events promote transgene decondensation. Interpretation of the transgene perturbation in cells expressing high level of EYFP-LacR-p150 is somewhat complicated. One possibility is that p150 has some intrinsic activity of chromatin unfolding, which may play a role in destabilizing the heterochromatin structure during DNA replication^33^. Alternatively, at high expression levels, exogenous p150 might drive formation of ectopic CAF-1 complexes with endogenous p60 and p48 that perturb the function of endogenous CAF-1.

Targeting HP1 to the transgene locus recruits p150/CAF1 and vice versa, suggesting mutually reinforcing localization of these two proteins. Our proposal that HP1 and CAF-1 cooperatively repress the transgene array might explain the observation that a N-terminally truncated mutant of human p150 lacking the HP1-binding domain impairs the maintenance of transcriptional gene silencing in a mouse cell line^34^. The p150/CAF-1 is required for a replication specific delivery of HP1 at pericentric heterochromatin sites during S phase^18^, suggesting a role distinct from its activity toward histones in nucleosome assembly. Studies of the budding yeast *S. cerevisiae* revealed that loss of CAF-1 causes defects in epigenetic gene silencing and a decline in heterochromatin spreading^35,36^. Studies in *Arabidopsis* showed that CAF-1 mutant has reduced cytological heterochromatin contents^37^, and is defective in maintaining the silent chromatin state^38^. Moreover, evidence has emerged that CAF-1 is required for the spatial organization of heterochromatin domains in pluripotent embryonic cells^39^. CAF-1 was also shown to regulate HP1-mediated epigenetic silencing and pericentric heterochromatin stability in Drosophila^40^. A recent study in fruit fly reported that maintenance of heterochromatin by the large subunit of the CAF-1 complex requires its interaction with HP1^41^. Taken together, these results suggest a conserved role for CAF-1 in heterochromatin formation and maintenance in eukaryotes.

The heterochromatic transgene array in our cell line resembles constitutive centromeric heterochromatin in its repetitive DNA content and association with HP1, H3K9me3, and DNA methylation. Yet the transgene sequences are completely different from the satellite DNA repeats found in centromeres, suggesting that heterochromatin is nucleated primarily based on epigenetic factors but not on specific DNA sequence. We propose a model that the repetitive nature of DNA sequences, which might confer distinctive bending and folding features, or the ability to produce dsRNA, is the key factor recognized by heterochromatinization machinery including histone HMT. The HMT then writes a marker on chromatin by methylating H3K9. HP1 localizes to the DNA repeats by reading the H3K9me mark and establishes mutually-reinforcing secondary interactions with p150/CAF-1 and other proteins, which consequently allow the chromatin to be compacted to a greater extent by forming characteristic higher-order chromatin domains. Taken together, our understanding of heterochromatinization at the repetitive transgene array provides important insights into how heterochromatic domains with large amount of DNA repeats are organized at chromosomal regions such as centromeres and telomeres.

## Methods

### Plasmids construction

The schematic of final constructs is shown as Supplementary Table S1 in the Supplemental Information. The constructs of pECFP-C1, pEYFP-LacR, pEYFP-LacR-VP16, phTet-On-VP16, phTet-On-Flag-VP16, phTet-On-Flag-NLS-VP16, phTet-On-Flag-NLS-ΔHP1 (equivalent to phTet-On-Flag-NLS-ΔVP16), phTet-On-Flag-HP1α were described previously^21^. All PCR amplifications were carried out with Pfu DNA polymerase (Stratagene). Mutations were introduced by PCR-based approach using cDNAs cloned in pBluescriptIIKS(−) (Stratagene). The mouse p150 cDNAs were digested by BsiWI/BamHI and subcloned into BsiWI/BamHI-digested pEYFP-LacR-VP16 or phTet-On-Flag-VP16, resulting in pEYFP-LacR-fused p150, p150L242N, p150ΔPMVVL or p150Δp60BD, and phTet-On-Flag-fused p150L242N or p150ΔPMVVL. The BsiWI (blunt-ended by Klenow)/BamHI-digested p150 cDNAs were subcloned into EcoRI (blunt-ended)/BamHI-digested pECFP-C1, resulting in pECFP-fused p150, p150L242N, p150ΔPMVVL and p150Δp60BD. The constructs of pECFP-p60 or p48 were made similarly as p150. The pSV2-p150 plasmid encoding p150 without any tag was generated by self-ligating NheI (blunt-ended)/HindIII (blunt-ended)-digested pSV2-ECFP-p150. pTet-On-TR was constructed by replacing VP16 of pTet-On (Clontech) with a PstI (blunt-ended)/BamHI-digested fragment encoding a ligand-binding domain of human TRβ (residues 201–456). The p16TRE-CMVm-luciferase plasmid was composed of 16 copies of TRE repeats followed by the minimal CMV promoter, a firefly luciferase cDNA and the β-globin intron.

### Cell culture, plasmid transfection, RNAi and fluorescence microscopy

Clone 2 cells and BHK cells were maintained as described previously^21^. Unless otherwise indicated, the cells used here were clone 2 cells. Dox and T3 were used at concentration of 1.0 µg/ml and 10 µM, respectively. Cells were transfected with plasmid using Lipofectamine 2000 (Invitrogen) and harvested for analyses at 16–24 hours later. Transfection of siRNA was performed by Lipofectamine 2000 or Oligofectamine (Invitrogen). To achieve optimal RNAi depletion, cells were treated with two consecutive siRNA transfections (45 nM) at 24 hours intervals followed by a third siRNA transfection (9.0 nM) together with plasmids as indicated in specific experiments. A mixture of two siRNA duplexes targeting the hamster p150 gene was used (Nippon EGT): sense-1, 5′-CGAGGACGAUGGUUUCUUUdTdT-3′, sense-1, 5′-AAAGAAACCAUCGUCCUCGdTdT-3′; sense-2, 5′-AAAGGGACCAGCAUAUCCUdTdT-3′, antisense-2, 5′-AGGAUAUGCUGGUCCCUUUdTdT-3′. A negative control siRNA duplex was used: sense, 5′-GAUCUGCUCGUACAAGAAUdTdT-3′, antisense, 5′-AUUCUUGUACGAGCAGAUCdTdT-3′. For fluorescence microscopy, cells were fixed in 4% formaldehyde in PBS for 1 hour and imaged using a Zeiss Axiovert 200 M inverted fluorescence microscope. The acquired images were processed using Adobe Photoshop.

### Cloning of a hamster p150 partial cDNA

Cloning of cDNA was performed as previously described^21^. The first-strand cDNA was transcribed by ReverTra Ace reverse transcriptase (Toyobo) from total RNA isolated from BHK cells, using a reverse primer corresponding to a human p150 cDNA. PCR amplification was carried out using Pfu DNA polymerase with a forward primer for human p150 primer and a reverse primer used in the first-stand cDNA transcription. The 372 bp amplified fragment was subcloned into the EcoRV site of pBluescriptIIKS(−) and sequenced (GenBank/EMBL/DDBJ accession number: AB264795).

### Quantitative real-time RT-PCR

As described previously^21^, real-time RT-PCR was performed in an iCycler (Bio-Rad) using a one-step QuantiTect SYBR Green RT-PCR Kit (Qiagen). The reverse transcription was carried out at 50 °C for 30 minutes, followed by an initial PCR activation step at 95 °C for 15 minutes. The subsequent PCR conditions were: 30 cycles of 15 secondes at 94 °C, 30 seconds at 56 °C and 30 seconds at 72 °C. The relative levels of CFP mRNA and p150 mRNA were normalized to the corresponding mRNA level of β-actin. The primers used were: hamster p150 forward 5′-CCATGGGTACCTGTCTGAGG-3′, reverse 5′-ATACACACCCCACTTGCACA-3′; hamster β-actin forward 5′-GTCGTACCACTGGCATTGTG-3′, reverse 5′-CCATCTCTTGCTCGAAGTCC-3′; CFP forward 5′-GGGCACAAATTTTCTGTCAG-3′, reverse 5′-AAAGCATTGAACACCCCAAG-3′.

### ChIP assay

ChIP assays were performed using a ChIP assay kit (Upstate) as we previously described^21^. Briefly, cells were cross-linked with 1% formaldehyde and lysed in sodium dodecyl sulfate (SDS) lysis buffer. Genomic DNA was fragmented into 300 to 500 bp in length by sonication of the cell lysates with a Bioruptor (Cosmo Bio). The pre-cleared supernatants were subjected to immunoprecipitation with antibodies against HP1ß^21^ or H3K9me3 (ab8898; Abcam). The immune complexes were recovered using the salmon sperm DNA/protein A agarose slurry and subsequently eluted with 1% SDS and 0.1 M NaHCO_3_. After reversal of the cross-links, the DNA was purified using a Qiaquick PCR Purification kit (Qiagen) and subjected to quantitative real-time PCR using SYBR Green Supermix (Bio-Rad) in an iCycler (Bio-Rad). The PCR cycling conditions were 3 minutes at 95 °C, followed by 40 cycles of 45 seconds at 94 °C, 30 seconds at 55 °C, and 30 seconds at 72 °C. The annealing temperature amplification of the ß-actin promoter was 64 °C. The primers used were: CMV promoter forward, 5′-GTCGACCGGGTCGAGGTAG-3′, reverse, 5′-GGGACAACTCCAGTGAAAAG-3′; hamster β-actin promoter forward, 5′-AATGCTGCACTGTGCGGCTA-3′, reverse, 5′-ACGCGGACTCGACAGTGGCT-3′.

### Immunoblotting

Western blotting analysis was performed as previously described^21^. The primary antibodies were rabbit antibodies against GFP (1:500 dilution, AB3080; Chemicon), human β-actin (1:2,500, ab8227; Abcam), mouse antibodies against Flag M2 (1:500, F3165; Sigma), human CAF-1 p150 (1:1,000, ab24746; Abcam). The secondary antibodies were horseradish peroxidase-conjugated donkey anti-rabbit IgG (1:2,500, NA934; Amersham Biosciences) and sheep anti-mouse IgG (1:7,500, NA931; Amersham Biosciences). ECL Plus reagents were from Amersham Biosciences. Chemiluminescence was detected using an LAS1000UV mini imager and quantified using the Image Gauge software (Fuji Film).

### Dual luciferase assay

Transfection of BHK cells were performed in a 96-well-plate. A total of 90 ng of p16TRE-CMVm-luciferase and 10 ng of phRL-TK (*Renilla* luciferase expression vector; Promega) were cotransfected into the cells with 50 ng of phTet-On-Flag-NLS-VP16 and 50 ng of phTet-On-Flag-fused cDNAs and incubated for 23 hours in the presence of Dox. Luciferase assays were performed using a Dual-Glo Luciferase Assay system (Promega) in a Wallac ARVO SX 1420 Multilabel Counter (Perkin Elmer Life Sciences) according to the manufacturer’s instructions. The *Renilla* luciferase activity was used to standardize the transfection efficiency.

## Electronic supplementary material

Supplementary Figure S1 and Table S1
