# Supplementary material for: HP1 cooperates with CAF-1 to compact heterochromatic transgene repeats in mammalian cells
Source: Sci Rep. 2018 Sep 20;8:14141. doi: 10.1038/s41598-018-32381-7 (PMC6147918; doi:10.1038/s41598-018-32381-7)
Supplement: Supplementary file 1 — Supplementary Figure S1 and Table S1 [file 41598_2018_32381_MOESM1_ESM.pdf]

Supplemental Information

HP1 cooperates with CAF-1 to compact heterochromatic transgene repeats in mammalian cells

Haiyan Yan[1, 2, *], Xingfeng Xiang[1, 2], Qinfu Chen[1], Xuan Pan[1], Hankun Cheng[1], Fangwei Wang[1, *]

Supplementary Figure S1. The original full-length blots related to Figure 2c.

Lane 1: Mock

Lane 2: VP16+ΔHP1

Lane 3: VP16+HP1αWT

Lane 4: VP16+HP1αI165E

Lane 5: VP16+HP1αW174A

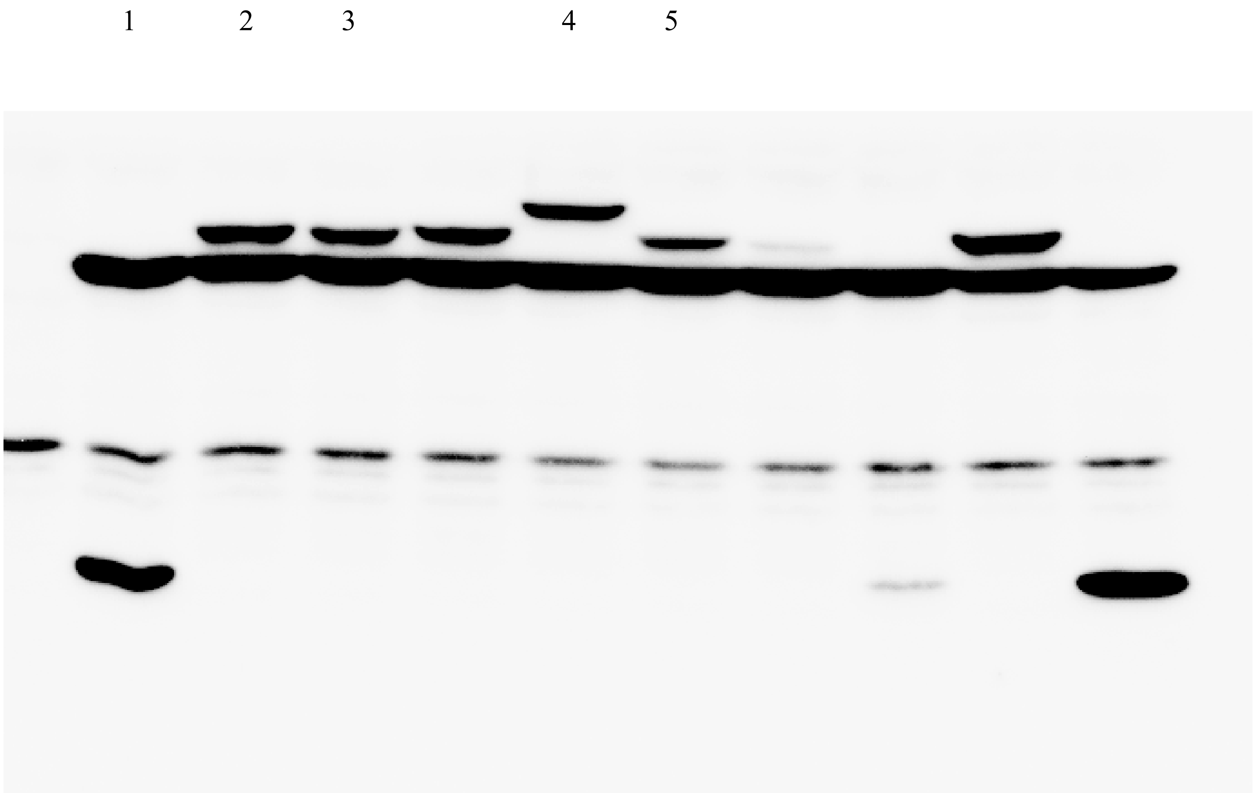

Supplementary Table S1. Schematics of plasmid constructs.

phTet-On-Flag	hrTetR	Flag		
pTet-On-TR	rTetR	TRβ		
phTet-On-VP16	hrTetR	VP16		
phTet-On-Flag-NLS-VP16	hrTetR	Flag	NLS	VP16
phTet-On-Flag-NLS-ΔHP1	hrTetR	Flag	NLS	
phTet-On-Flag-HP1α	hrTetR	Flag	HP1	
phTet-On-Flag-p150	hrTetR	Flag	p150	
pEYFP-LacR	EYFP	LacR		
pEYFP-LacR-VP16	EYFP	LacR	VP16	
pEYFP-LacR-p150	EYFP	LacR	p150	
pECFP-p150	ECFP	p150		
pECFP-p60	ECFP	p60		
pECFP-p48	ECFP	p48		
pSV2-p150	pSV2	p150		
p16TRE-CMVm-Luciferase	16xTRE repeats	$Pcmv_m$	Firefly luciferase	
